# The structure of slip-pulses and supershear ruptures driving slip in bimaterial friction

**DOI:** 10.1038/ncomms11787

**Published:** 2016-06-09

**Authors:** Hadar Shlomai, Jay Fineberg

**Affiliations:** 1 The Racah Institute of Physics, The Hebrew University of Jerusalem, Givat Ram, Jerusalem 9190401, Israel

## Abstract

The most general frictional motion in nature involves bimaterial interfaces, when contacting bodies possess different elastic properties. Frictional motion occurs when the contacts composing the interface separating these bodies detach via propagating rupture fronts. Coupling between slip and normal stress variations is unique to bimaterial interfaces. Here we use high speed simultaneous measurements of slip velocities, real contact area and stresses to explicitly reveal this bimaterial coupling and its role in determining different classes of rupture modes and their structures. We directly observe slip-pulses, highly localized slip accompanied by large local reduction of the normal stress near the rupture tip. These pulses propagate in the direction of motion of the softer material at a selected (maximal) velocity and continuously evolve while propagating. In the opposite direction bimaterial coupling favors crack-like ‘supershear' fronts. The robustness of these structures shows the importance of bimaterial coupling to frictional motion and modes of frictional dissipation.

## Abstract (web summary)

Friction commonly involves different material types (bimaterials) at their sliding interface. Here, in laboratory experiments Shlomai and Fineberg reveal effects uniquely due to biomaterial coupling, with slip-pulses and crack-like supershear fronts dominating opposing propagation directions.

## Graphical abstract

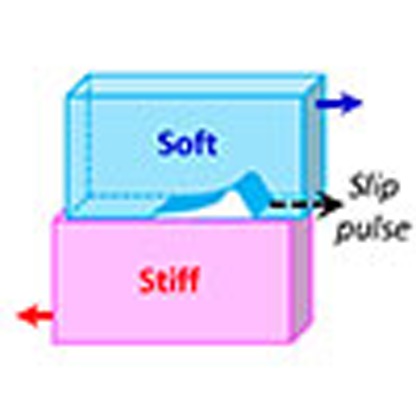

Most studies of frictional sliding have considered homogeneous systems involving sliding bodies composed of the same material^1^,^2^,^3^,^4^. Within homogenous interfaces, the rupture fronts that generally mediate the onset of friction are closely related to classic shear cracks^3^, which are characterized by near-tip singularity and extended slip duration. Spatially localized slip, pulse-like ruptures, may also occur along homogenous interfaces^5^,^6^,^7^,^8^, but are generally associated with the form of the friction law; occurring when frictional resistance is markedly reduced near a rupture tip. Examples include extreme velocity weakening^5^,^9^ or flash heating^9^.

The existence of a bimaterial interface can bring about qualitative differences in how contact points detach^5^,^8^,^10^,^11^,^12^,^13^,^14^,^15^,^16^,^17^,^18^,^19^,^20^,^21^. In homogenous systems, due to the symmetry of the system, stress-field components of both crack-like and pulse-like ruptures are either symmetric (Δ*σ*_*ij*_(−*y*)=Δ*σ*_*ij*_(*y*)) or anti-symmetric (Δ*σ*_*ij*_(−*y*)=−Δ*σ*_*ij*_ (*y*)). In contrast, any slip within a bimaterial interface will break the stress symmetry across the interface. One important result of this is that local values of normal stress variations at the interface are theoretically expected to couple to interface slip. This ‘bimaterial coupling' is an elastodynamic coupling and is independent of specific properties of the friction law at the interface. Bimaterial coupling is strongest at the rupture tip, where stresses are maximal, and intensifies strongly with both the ratio of sound wave velocities^10^,^13^ of the contacting materials (‘material contrast')^5^,^13^ and with the rupture front velocity^10^,^19^, *C*_f_. The sign of the coupling depends on the front propagation direction. Ruptures propagating in the direction of motion of the more compliant material, which we will call the ‘positive' direction, can radically decrease the normal stress^10^,^12^,^19^, *σ*_*yy*_, near the rupture tip. Therefore, in the positive direction, frictional resistance locally decreases, enhancing slip. Frictional resistance may theoretically vanish as *C*_f_ (*t*) approaches its limiting velocity, the generalized Rayleigh wave speed, *C*_GR_, for low material contrasts and the shear wave velocity of the compliant material, 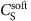, for higher material contrasts (Methods). This elastodynamic reduction in *σ*_*yy*_ is predicted to lead to distinct slip-pulses^10^,^12^ characterized by slip that is strongly localized around rupture tips and driven by highly localized reduction of *σ*_*yy*_. In the opposite ‘negative' direction, where motion of the compliant material is opposite the rupture direction, the sign of the coupling is reversed for sub-sonic (*C*_f_<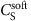) ruptures^8^,^17^ and *σ*_*yy*_ near rupture tips increases. Only for ‘supershear' ruptures (*C*_f_>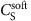) does bimaterial coupling enhance slip when ruptures propagate in the negative direction^8^,^17^,^18^,^21^. The limiting velocity in the negative direction is therefore predicted to be slightly below the *P*-wave velocity of the softer material^8^,^17^,^21^, 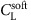.

Relatively few experiments have focused on bimaterial frictional rupture^6^,^7^,^22^,^23^. Experiments using low material contrast^7^,^23^ observed strong directionality; ruptures in the positive direction were limited by *C*_GR_, whereas in the negative direction sub-shear ruptures were seen to transition to supershear. Follow-up experiments^22^ also observed transitions to the fast 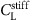 in the positive direction. In systems with strong material contrast, single-point particle velocity and normal displacement measurements provided evidence for transient opening^6^. These experiments, however, measured continuous spatially extended sliding similar to rupture of homogeneous interfaces in the same set-up^7^,^17^,^23^, in apparent contradiction to claims of pulse-like ruptures. This apparent confusion may be due to the explosive triggering used in all of the above experiments (point explosions or projectile impact), whose effect on rupture mode selection is not fully understood^17^. No direct measurements of the stress fields driving rupture exist.

Many aspects of bimaterial-induced ruptures are controversial; questions of slip-pulse stability^8^,^15^,^19^,^20^,^21^,^24^, mode and velocity selection^7^,^8^,^12^,^16^,^17^,^25^ and the possibility of separation at the interface^7^,^12^,^15^,^19^,^21^ abound. As both slip-pulses and crack-like modes may coexist along bimaterial interfaces^6^,^12^,^17^,^25^, which of these dominates is an important open question^8^.

Here we study spontaneously nucleated slip along bimaterial interfaces under quasi-static loading, measuring the real contact area and full strain tensor near the interface at high speeds. These measurements explicitly demonstrate the unique effects of bimaterial coupling and reveal structures that are characteristic only to this coupling; slip-pulses propagating exclusively at 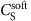 in the positive direction and solely crack-like propagation in the negative one that are dominated by supershear modes.

## Results

### Experimental system

**Figure 1:**
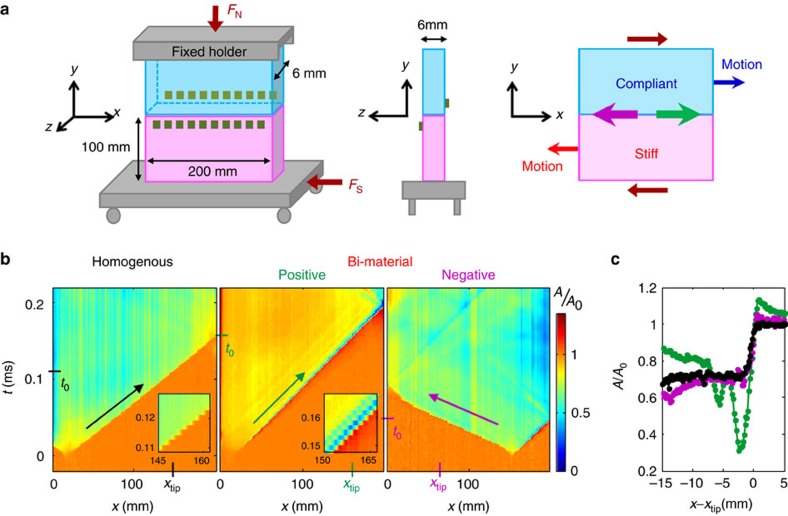
Experimental system and comparison of rupture profiles. (**a**) Twenty rosette strain gauges (green squares) are mounted ≈2 mm above and beneath the frictional interface (left), on opposing block faces (centre). Green (purple) arrows define positive (negative) rupture propagation directions as parallel (anti-parallel) to the direction of the motion of the compliant material. (**b**) The real contact area, *A*(*x*,*t*) (normalized before nucleation at *t*=0), along the 200 mm quasi-1D interface. Insets: magnified sections of *A*(*x*,*t*). (**c**) *A*(*x*−*x*_tip_) measured around rupture tip locations, *x*_tip,_ denoted in **a**; (black) PMMA on PMMA (homogenous system), *C*_f_=0.94*C*_R_, *F*_N_=3,402 N, (green) Polycarbonate (PC) on PMMA (bimaterial system) for rupture in the positive direction, *C*_f_=0.99*C*_S_^soft^, *F*_N_=3,863 N, and (purple) leftward supershear rupture in the negative direction, *C*_f_=0.92*C*_L_^soft^, *F*_N_=3,553 N. Negative direction propagation was reversed for comparison.

Our experimental system consists of both homogeneous and bimaterial interfaces composed of, respectively, two PMMA (polymethylmethacrylate) blocks or polycarbonate (PC) sliding on PMMA (Fig. 1a). The material wave velocities are: 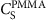
=1,345±10 ms^−1^, 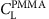
=2,330±10 ms^−1^, 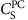
=932±20 ms^−1^ and 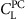
=1,690±20 ms^−1^, providing a ∼40% material contrast. 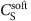
=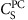 is the expected limiting velocity in the positive direction. Both blocks are first compressed with a normal force, 2,000<*F*_N_<6,000 N. Slip is triggered quasi-statically either by increasing the shear force *F*_S_ at fixed *F*_N_ or, alternatively, by fixing *F*_S_ and reducing *F*_N_ (Methods). The applied values of *F*_S_ and *F*_N_ had no significant effect on our results. Throughout the experiments, we performed continuous optical measurements of the real contact area, *A*(*x*,*t*), along the entire interface with (*x* × *z*) spatial resolution 1,280 × 8 pixels at 580,000 frames per second. *A*(*x*,*t*) (averaged over *z*) are normalized relative to *A*_0_(*x*), defined as *A*(*x*) at ∼10 ms before rupture onset. The rupture front location, *x*_tip_, is defined as the point where *A*(*x*_tip_)=0.95·*A*_0_(*x*). *C*_f_ (*t*) was obtained from *x*_tip_(*t*). Simultaneous measurements of the strain tensor, *ɛ*_*ij*_(*t*) were performed at 20 locations along and ≈2 mm above and beneath the interface with each strain component measured at 10^6^ samples per second. For rupture fronts propagating with constant velocity *ɛ*_*ij*_(*x*,*t*)=*ɛ*_*ij*_(*x*−*C*_f_*t*). Using this^3^, we converted *ɛ*_*ij*_(*t*) to spatial measurements *ɛ*_*ij*_(*x*−*x*_tip_), stresses *σ*_*ij*_(*x*−*x*_tip_) (plane stress conditions), and particle velocities 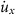
=**−**Δ*ɛ*_*xx*_*C*_f_ (*u* is the displacement field). Ruptures in the positive (negative) direction will be presented as propagating from left to right (right to left), as defined in Fig. 1a.

### Contact area profiles

Figure 1b compares real contact area, *A*(*x*,*t*), measurements of typical ruptures propagating near their theoretical asymptotic velocities; the Rayleigh wave velocity, *C*_R_, for homogenous systems, 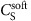 for bimaterial fronts in the positive direction, and supershear velocities approaching 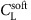 in the negative direction. Figure 1c presents corresponding typical contact area profiles *A*(*x*−*x*_tip_) at specific times, *t*_0_.

*A*(*x*,*t*) is a quantity that reflects the instantaneous interface strength and is determined by both *σ*_*yy*_(*x*,*t*) and the age of the contacts. During rupture front propagation, contacts are broken at the rupture tip and reform once the front passes. Along homogenous interfaces, *A*(*x*−*x*_tip_) drops like a step function from *A*_0_(*x*) to instantaneous residual values of 0.6−0.8*A*_0_(*x*) that result from the fractured/broken contacts. Residual values remain constant for the duration of a slip event, which is shorter than the logarithmic aging time^2^ needed to restore *A*_0_(*x*).

Along the positive direction in bimaterial systems, *A*(*x*,*t*) have much richer dynamics of contact formation and separation. Before rupture arrival, *A*(*x*) increases by 15–30%, suggesting an initial compression. As the rupture tip passes, *A*(*x*) can drop to as low as 0.03*A*_0_(*x*). In contrast to homogeneous systems, within a few mm's from *x*_tip_, this large drop in *A*(*x*) is followed by a rapid (dynamic) increase to residual values of 0.7–0.9*A*_0_(*x*) (0.9*A*_0_(*x*) in Fig. 1b. This re-strengthening takes place over times ≈10 μs which are orders of magnitude less than the aging recovery time in homogeneous systems^2^. For supershear fronts along the negative direction in the bimaterial system, *A*(*x*) drop like step functions to 0.6–0.8*A*_0_(*x*), with slight transient variations of *A*(*x*) reminiscent of the strong bimaterial effect in the positive direction.

**Figure 2:**
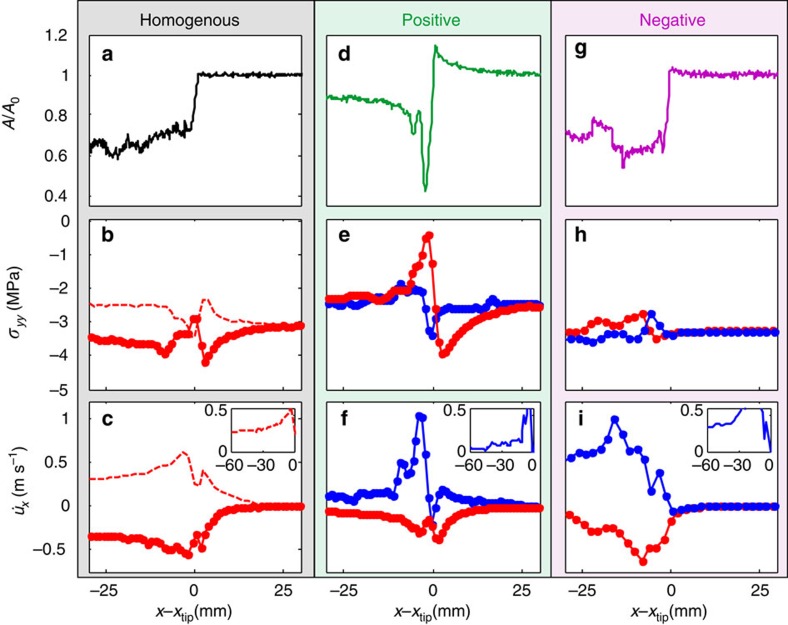
Contact area and normal stress and particle velocity measurements indicate slip-pulses in the positive direction and supershear cracks in the negative one. Comparisons of contact area, *A*(*x*,*t*) (**a**,**d**,**g**), normal stresses, *σ*_*yy*_(*t*) (**b**,**e**,**h**), and particle velocities, 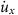, (**c**,**f**,**i**) for the ruptures presented in Fig. 1b. Signs of 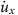 correspond to the motion directions denoted in Fig. 1a. Line colours represent measurements within the different blocks: red lines denote measurements within the stiff material (PMMA) and blue measurements within the compliant material (PC). (**a**–**c**) Homogenous interface, *x*_tip_=109 mm: (red dashed line) top block, (solid line) bottom block. Bimaterial interfaces, (**d**–**f**) positive direction, *x*_tip_=125 mm and (**g**–**i**) negative direction, *x*_tip_=87 mm. Ruptures along the positive direction are characterized by highly localized signals compared with those in the homogenous and negative directions. Insets in **c**,**f** and **i**: 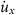 measurements for −60<*x−x*_*tip*_<0 demonstrate slip localization solely in the positive direction. Line colours in **d** and **g** correspond to the rupture propagation directions denoted in Fig. 1.

The unique behaviour of *A*(*x*,*t*) in the positive direction suggests that the rapid variations of *A*(*x*) echo local variations of *σ*_*yy*_(*x,t*) as predicted^5^,^8^,^10^,^12^,^13^,^17^. These strong correlations are explicitly presented in Fig. 2, where we compare direct measurements of *A*(*x*,*t*), *σ*_*yy*_ and 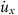 surrounding the rupture tip. As we measure 2 mm from the interface, the magnitudes of 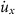 and Δ*σ*_*yy*_ are lower bounds of their values on the interface. For singular fronts these should significantly exceed those of Fig. 2.

### Comparison of characteristic structures

We first consider the homogeneous system in Fig. 2a–c where *σ*_*yy*_ and 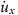 correspond to (shear) crack-like (extended slip) ruptures^3^. Normal stress variations from the initial value, Δ*σ*_*yy*_, are clearly anti-symmetric. This is consistent with Δ*σ*_*yy*_=0 at the interface with the drop in *A*(*x*,*t*) solely due to fractured contacts. As expected for shear cracks, 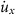 have long tails beyond the rupture tip (Fig. 2c inset) and increase with both *C*_f_ and *F*_N_ (ref. 26).

We now turn to bimaterial ruptures in the positive direction (Fig. 2d–f). Δ*σ*_*yy*_ and 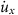 are highly asymmetric in both their structure and amplitude. As Δ*σ*_*yy*_ in the stiffer material have much larger amplitudes than in softer material, we consider this signal as representative of the normal stress variations at the interface. As the front approaches, *σ*_*yy*_ undergoes compression corresponding to the increase of *A*(*x*) before rupture. Both *σ*_*yy*_ and *A*(*x*) at the rupture tip evolve with the distance from the rupture tip. At the rupture tip the reduction in both of these quantities is extreme and both can approach zero. The reductions of *σ*_*yy*_ and *A* are highly localized in space and time compared with the homogenous system. Within ∼10 μs (∼10 mm) after the rupture tip passes, *σ*_*yy*_ is dynamically restored to its initial value. Similar behaviour is exhibited by *A*(*x*−*x*_tip_), although the recovery of *A*(*x*−*x*_tip_) is incomplete due to broken/separated contacts^2^. In the same ∼10 μs interval, all of the slip takes place; 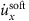 is dominant and large (can surpass 2 ms^−1^), highly peaked immediately following the rupture tip and 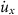
**→** 0 once *σ*_*yy*_ is dynamically restored.

When considered together, the structures, magnitudes and symmetries of Δ*σ*_*yy*_ and 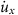 provide clear evidence for the coupling between slip and *σ*_*yy*_ unique to bimaterial interfaces. The large variations over such short durations reveal that propagation in the positive direction is governed by slip-pulses driven by strong bimaterial coupling. In the negative direction, Δ*σ*_*yy*_ and 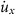 are not localized in space and slowly decrease (Fig. 2i inset) after attaining peak values. Δ*σ*_*yy*_ are an order of magnitude smaller than in the positive direction. While both Δ*σ*_*yy*_ and 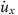 possess pronounced asymmetry, bimaterial supershear ruptures are not pulse-like but, instead, involve crack-like propagation in the negative direction.

### Slip-pulse evolution and velocity selection

**Figure 3:**
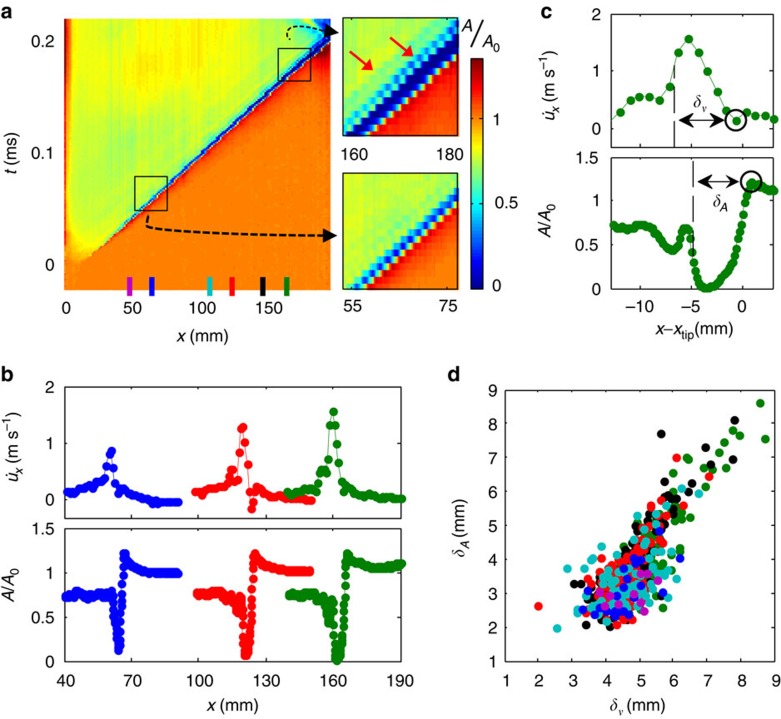
Slip-pulses continuously evolve with propagation. (**a**) *A*(*x*,*t*) of a typical front in the positive direction. Right panels are close-ups of *A*(*x*,*t*) in the marked areas. Red arrows denote secondary and tertiary slip-pulses that follow the main slip-pulse which are absent during the earlier (55–75 mm) propagation. (**b**) 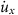
(*x*) (top) and *A*(*x*) (bottom) profiles along the interface. Each profile is displayed around *x*_tip_ locations coinciding with the strain gauge locations (coloured lines) denoted in **a**. (**c**) Close-ups of 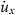 (*x*−*x*_tip_) (top) and *A*(*x*−*x*_tip_) (bottom) for *x*_tip_=165 mm. We define pulse widths; *δ_v_* is defined from the point where 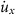 becomes positive (circle), to 2/3 of the difference between maximal and residual values (Methods). *δ*_*A*_ is defined from the point of maximal compression of *A*(*x*−*x*_tip_) (circle) to 1/3 of the difference between its residual and minimal values. (**d**) *δ*_*A*_ versus *δ*_*v*_ for 131 fronts for 2,000<*F*_N_<6,000 N. Colours: locations denoted in **a**.

Figure 3a displays the typical evolution of *A*(*x*,*t*) for a propagating slip-pulse. The slip-pulses continuously evolve; we have never observed steady-state propagation in the positive direction. Figure 3b shows that the magnitudes of both 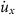 and the variations of *A*(*x*,*t*) increase significantly with propagation distance. In addition, as a slip-pulse evolves, its pulse width, both in 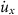 and *A*(*x*,*t*), can more than double (Fig. 3d) with propagation distance. Figure 3d demonstrates the rough proportionality of the widths of the particle velocity, *δ*_*v*_, and contact area, *δ*_*A*_ (Fig. 3c), over a wide range of loading and nucleation conditions. Deviations from proportionality for narrow pulses may result from our finite (∼1 μs) temporal resolution. *δ*_*A*_ always precedes *δ*_*v*_. This consistent phase shift may either be a characteristic feature of slip-pulses or may result from the fact that *δ*_*v*_ is measured 2 mm above the interface while *δ*_*A*_ is measured on the interface.

A characteristic feature of the observed slip-pulses is the appearance of evolving secondary pulses that follow the main rupture. Secondary pulse appearance is highlighted in Fig. 3a. Secondary pulses are responsible for the fine structure evident in both in 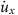 and *A*(*x*,*t*) in Fig. 3b,c and can reach half of the main pulse amplitude.

The results of Fig. 3 suggest that no real slip-pulse stability may exist; 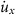, *σ*_*yy*_ and *A*(*x*,*t*) constantly evolve with propagation distance. This evolution could be due to the asymptotic approach of *C*_f_ to 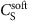 in the positive direction or, alternatively, result from an intrinsic lack of stability of this mode. Such an instability, known as the Adam's instability^13^,^21^,^24^ has been predicted; slip-pulses are expected to sharpen^12^,^17^,^24^ and eventually break up with evolution^12^,^19^. This may also explain the nucleation of the secondary pulses observed here.

**Figure 4:**
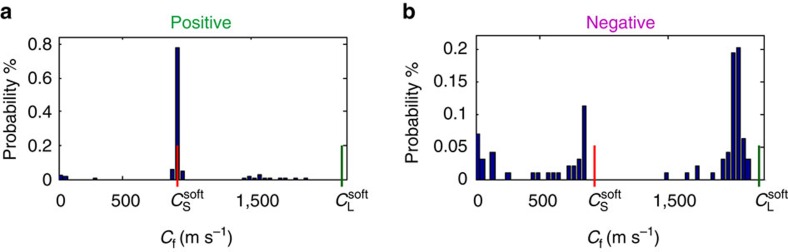
Rupture velocities are sharply selected in the positive direction whereas supershear dominates negative direction ruptures. (**a**) Histogram of 213 steadily propagating (Methods) ruptures in the positive direction for various experimental loading conditions and 2,000<*F*_N_<6,000 N. (**b**) Histogram of 98 steadily propagating ruptures in the negative direction, conditions as in **a**. Green and red lines denote, respectively, *C*_L_^soft^ and *C*_S_^soft^. Note the sharp selection (88% of all primary ruptures) at *C*_S_^soft^ in the positive direction whereas *C*_S_^soft^ is never observed in the negative one.

Figure 4 unambiguously shows that rupture velocity distributions in opposite propagation directions are entirely different, as both predictions^8^,^10^,^12^,^13^,^17^,^21^ and previous experiments^22^,^23^ have suggested. In the positive direction, nearly all ruptures have a sharp well-defined velocity, *C*_f_=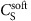. In this direction supershear fronts, over a wide range of velocities, are rarely observed. All of these nucleate only as secondary ruptures ahead of the concurrently propagating main rupture at 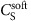.

In the negative direction supershear fronts dominate propagation. Their velocities are concentrated near *C*_f_=0.91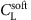. They nucleate directly, without being preceded by a well-defined stage of sub-Rayleigh rupture. The distinction between the smooth transition to supershear in the negative direction and the transition as a secondary nucleation in the positive direction is consistent with recent simulations^27^ that considered bimaterial coupling. In the negative direction we sometimes observe ruptures at sub-Rayleigh velocities. Their structure appears similar to homogeneous crack-like ruptures and, significantly, *C*_f_ never approaches 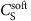 in the negative direction.

The rupture velocity values and asymmetric distributions in the different directions (Fig. 4) provide further evidence for strong bimaterial coupling. Particularly telling observations are the sharp selection in the positive direction at *C*_f_=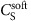 with near-total disappearance of lower sub-shear velocities and the observation that *C*_f_ never approaches 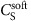 in the negative one. These are theoretically expected results^13^; in the positive direction bimaterial coupling is maximal at 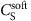 when *σ*_*yy*_ approaches (Fig. 2e) near-zero values, whereas increased *σ*_*yy*_ at the tip is expected when propagating in the negative direction.

## Discussion

The unique structures and features that characterize ruptures along bimaterial interfaces in our experiments indicate a clear dichotomy of the dominant propagation modes along bimaterial interfaces; unambiguous slip-pulses with a single sharply selected velocity in the positive direction and supershear ruptures with crack-like features in the negative one. These observations establish the importance of bimaterial coupling in driving interface dynamics. We have explicitly shown that 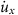, *A*(*x*,*t*) and *σ*_*yy*_ strongly couple to produce these diverse structures, as predicted by bimaterial coupling with no need to invoke effects due to friction laws.

Due to the inherent generality of bimaterial interfaces, we expect that these results may have important implications for our fundamental understanding of the onset of frictional motion and where and how frictional dissipation occurs. The properties of bimaterial ruptures are of particular interest in earthquake dynamics^28^. While it is clear that natural faults are considerably more complex than the ‘simple' frictional interfaces studied here, the general nature of the features described above leads us to expect them to persist in faults bounded by different rock types. For example, the detailed structure of the stress fields described in our results supports observations of the coupling between earthquake directionality and off-fault damage^29^.

## Methods

### System and material properties

Our experiments were conducted using two sets of blocks. For the homogenous interface we used two poly(methylmethacrylate) (PMMA) blocks of dimensions 220 × 100 × 5.5 mm (top block) and 200 × 100 × 5.5 mm (bottom block) in the *x*, *y* and *z* direction, respectively (Fig. 1a). For the bimaterial interface we used a polycarbonate (PC) block of dimensions 197 × 100 × 5.8 mm sliding on the 220 × 100 × 5.5 mm PMMA block. The contact faces of the blocks were diamond-machined to optical flatness.

Material shear, *C*_S_, and longitudinal, *C*_L_, wave speeds were obtained by measuring the time of flight of 5 MHz ultrasonic pulses, yielding 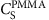
=1,345±10 ms^−1^, 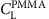
=2,700±10 ms^−1^, 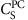
=932±20 ms^−1^ and 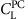
=2,220±20 ms^−1^. Due to the small wavelength of the ultrasonic pulses used compared with the dimensions of the measurement set-up, the measured *C*_L_ correspond to plane strain conditions (*ɛ*_*zz*_=0). The small *z* dimension of the experimental set-up implies plane stress (*σ*_*zz*_=0) conditions in our experiments. Using the above measured velocities, *C*_L_ for plane stress were calculated to be 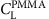
=2,330±10 ms^−1^ and 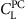
=1,690±20 ms^−1^. The corresponding Rayleigh wave speed of the homogenous system is: 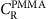
=1,237±10 ms^−1^. The generalized Rayleigh wave speed, *C*_GR_, of a bimaterial interface is the speed where a disturbance, confined to the interface region, will propagate with no attenuation. *C*_GR_ is not defined for all values of the material contrast^5^,^10^,^21^. This is the case for the PMMA–PC system. In such cases, the limiting velocity in the preferred direction is predicted to be the slower shear wave velocity, 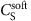
=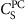.

The wave speeds and the mass density, *ρ*^PMMA^=1,170 kg m^−3^ and *ρ*^PC^=1,200 kg m^−3^, and the wave speed measurements yield dynamic values for the Poisson ratio of *ν*^PMMA^=0.33 and *ν*^PC^=0.39 and Young's moduli of 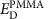
=5.6 GPa and 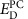
=2.9 GPa. Note that the values of E are significantly different from the static values 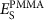
=3 GPa and 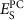
=2.4 GPa. This difference is due to viscoelastic behaviours of PMMA^30^ and PC.

### Loading application

In the experimental system, the top block was clamped at its top edge, while the bottom block was rigidly mounted at its bottom edge in a stiff low-friction linear translational stage. Both blocks were first compressed with a normal force which was varied between experiments throughout the range 2,000<*F*_N_<6,000 N (∼2<*σ*_*yy*_<5 MPa). External shear loads, *F*_S_, were then applied to the stiff translational stage which was constrained in its movement only by the frictional resistance at the interface with the top block. In this way, *F*_S_ was spatially distributed along the entire length of the interface. Both *F*_N_ and *F*_S_ were continuously monitored throughout the experiment by means of S-Beam load cells (of stiffness 10^6^–10^7^ N m^−1^) in series with the loading apparatus. An optional rigid stopper of cross-section 1 cm^2^ could be applied to the top block at *x*=0 mm, at a controllable height *h*, to constrain motion of this edge in the *x* direction and control torqueing. The application of the stopper thus introduced some elements of edge loading.

To explore a range of external loading conditions, the experiments were conducted using two distinct ways to trigger rupture nucleation: (1) *F*_S_ were applied to the system quasi-statically, at fixed *F*_N_, at loading rates between 4 and 15 N s^−1^ until slip initiated. With this triggering method, the ruptures were usually nucleated along the quarter of the interface close to *x*=0 mm, either as a result of the edge loading or reduced local normal force resulting from induced torques. (2) At the completion of a sequence of slip events, the residual *F*_S_ was kept fixed, and *F*_N_ was reduced at loading rates between 40 and 60 N s^−1^, resulting in spontaneous rupture nucleation. The last triggering method yielded a wider distribution of nucleation locations along the interface.

For both triggering methods, ruptures would simultaneously nucleate in both directions. The rupture propagation mode (positive or negative) of the longest rupture could be controlled by vertically inverting the compliant and stiff blocks. Our choice of whether the compliant (stiff) block was mounted on the top or bottom produced, as expected, no overall difference in our results. We note that while rupture events occurred while either *F*_S_ or *F*_N_ were modified, the changes in *F*_S_ or *F*_N_ were sufficiently slow so that their values were constant during the (100–200 μs) rupture propagation period.

### Real contact area measurements

Changes in the real contact area along the entire interface were measured by an optical method based on total internal reflection. Basic principles are presented in detail elsewhere^3^,^31^,^32^. A sheet of light, incident on the frictional interface at an angle well beyond the critical angle for total internal reflection, is reflected everywhere except at the contact points. This yields an instantaneous transmitted light intensity that is roughly proportional to *A*(*x*,*z*,*t*) over the entire (*x* × *z*) 200 × 5.5 mm interface. The transmitted light is continuously imaged (at a spatial resolution of 1,280 × 8 pixels) at 580,000 frames per second using a high speed camera, Phantom v711 at 12 bit accuracy. Data acquisition is continuous. The data are temporarily stored in a circular buffer large enough to acquire 7–13 ms of data, both before and after each event. The frictional interface is quasi-one-dimensional (1D), as its width (*z* direction), 5.5 mm, is much smaller than other dimensions of the block. The simultaneous measurements of *A*(*x*,*t*)=<*A*(*x*,*z*,*t*)>_*z*_ along the entire 1D interface are obtained by averaging of the acquired images over the 8 pixels in the *z* direction. We use a high power LED (CBT-120) as our illumination source of noncoherent light (Methods in Svetlizky & Fineberg^3^). The noise level after integration is ≤1% of the signal.

When the contact area at a point drops to only few per cent of its initial value (during a slip event when the rupture propagates in the preferred direction) the transmitted light is comparable to the background (due to scattered light at the interface). As a result, the values of contact area measurements quoted in the paper are maximal values. This implies that the two faces may actually (temporarily) completely detach from one another—that is, the interface may indeed separate within a slip-pulse in the preferred direction.

### Rupture front velocity *C*_f_ calculation

The rupture front location, *x*_tip_, is defined as the point where *A*(*x*_tip_)=0.95·*A*_0_(*x*). *C*_f_(*t*) is obtained from *x*_tip_(*t*). Our precision in determining in *C*_f_(*t*) depends on our 200 μm uncertainty in *x*. We consider ‘instantaneous' values of *C*_f_(*t*) as velocity values determined over 10 mm intervals. For these values of *C*_f_(*t*) our resolution varies between 1 and 2%.

We define steadily propagating ruptures with velocity *C*_f_ as ruptures having no clear tendency to accelerate or decelerate. Within these intervals instantaneous measurements of *C*_f_(*t*) change by <30 ms^−1^ while traversing distances of at least 50 mm. This is ≤ 1–3% of the velocity for the supershear and 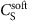 fronts presented in this study.

### Strain measurements

We use miniature Kulite B/UGP-1000-060-R3 rosette strain gauges for local strain measurements. Twenty such strain gauges are mounted along and ∼2 mm above and beneath the frictional interface, on opposing block faces (Fig. 1a). Each rosette strain gauge is composed of three independent active regions (each 0.4 × 0.9 mm in size)—two of these are oriented at ±45° relative to the third, which is oriented normal to the interface. The individual gauges are separated by 0.55 mm in the *x* direction. During rapid rupture propagation, this distance induces a small time delay ∼0.55/*C*_f_ ∼0.5 μs between the components. This was taken into account for the proper calculation of *ɛ*_*ij*_(*t*). The strain gauges have a slightly non-linear gauge factor response of Δ*R*/*R*=4,689*ɛ*^2^+110.1*ɛ*, which was also taken into account. All strain signals (60 channels) are amplified (gain=11, ∼1 MHz bandwidth) and simultaneously acquired to 14 bit accuracy by an ACQ132 digitizer (D-tAcq Solutions Ltd) at a 1 MHz rate. This leads to a sensitivity of ∼2μStrain in *ɛ*_*ij*_(*t*) measurements. As the signals are of order ∼1mStrain this provides a 0.2% uncertainty in *ɛ*_*ij*_.

As shown in Fig. 1a, the strain gauges were mounted on opposing faces of the two blocks (that is, at points [*x*,*y*,*z*]=[*x*,+2 mm,0] and [*x*,−2 mm, 5.5 mm]). Measured dynamic values (rapid variations) of *σ*_*xy*_ (*t*) on both sides of the interface, (*σ*_*xy*_ is the only continuous quantity expected across the interface) were in good agreement with <5–10% error. As a result, all dynamic measurements of *ɛ*_*ij*_ (*t*) reliably reflect the dynamic 1D signal along the interface.

The small differences in the block widths (<0.5 mm) and experimental alignment limitations, at times, resulted in discrepancies in *static* values of *ɛ*_*ij*_ (*t*<0) that were measured along opposing sides of the interface. We found that, despite these discrepancies, the average of opposing strain gauges agreed well with the mean value of the normal stress along the interface, as determined by dividing the applied normal force, *F*_N_, by the interface area. The last was used as the static value in the calculation of normal stress variations Δ*σ*_*yy*_(*t*)=*σ*_*yy*_(*t*)−*σ*_*yy*_(*t*<0) in the text.

As our evaluation of *σ*_*yy*_(*t*) and the slip velocity is done via strain gauges located 2 mm from the interface, our measurements only provide us with an estimate of these values on the interface. In the paper, we considered only the dominant signal in the two blocks; the softer material's (PC) signal for 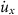 and the stiffer material's (PMMA) for *σ*_*yy*_. Estimates of these signals on the interface (for example, by averaging the two signals) only slightly alters their maximal amplitudes. Summing opposing signals was not performed when we considered stress or strain variations, however, because any uncertainty in the *x* location of opposing strain gauges (due to slip throughout an experiment) could bring about phase differences in opposing signals. Since these signals are rapidly varying quantities, any uncertainty in their relative phase could have induced large measurement errors.

For steadily moving rupture fronts *ɛ*_*ij*_(*x*,*t*)=*ɛ*_*ij*_(*x*−*C*_f_*t*). Using this, we converted *ɛ*_*ij*_(*t*) to spatial measurements *ɛ*_*ij*_(*x*−*x*_tip_) (Methods in Svetlizky & Fineberg^3^), stresses, *σ*_*ij*_(*x*−*x*_tip_) (assuming plane stress conditions) and particle velocities 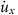
(*t*)=−*ɛ*_*xx*_(*t*)˙*C*_f_(*t*).

### Determination of pulse widths *δ*_*v*_ and *δ*_*A*_

We defined the widths of the slip velocity and contact area pulses by, respectively, *δ*_*v*_ and *δ*_*A*_, as follows (Fig. 4c). Slip initiation is identified with the point when the velocity becomes positive as a rupture passes. To avoid effects of secondary pulses (see inset Fig. 4a) on the determination of these widths, we defined the end of the *δ*_*v*_ when the slip velocity relaxed to two thirds of the difference between the maximal 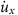 and its final residual value. Final residual values were defined as the average value in the range of 20–25 mm behind the rupture's tip. This range is sufficiently far to enable the large variations of 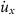 and *σ*_*yy*_ to relax.

In the same way, *δ*_*A*_ was defined from the point of maximal compression in *A*,before the reduction, to a third of the difference between the residual value (at 20–25 mm behind the rupture tip) of *A* and its minimal value.

The synchronization of 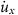
(*t*), measured by the strain gauges, together with the *A*(*x*,*t*), measured by the fast camera, requires precise knowledge of strain gauge locations. These were found using a reference image at the beginning of each experiment. The error in determining the locations is about ∼0.4–1 mm. This is smaller than the average phase shift between *δ*_*v*_ and *δ*_*Α*_ which correspond to∼1.5–2 mm.

### Data availability

The authors declare that the data supporting the findings of this study are available within the article.

## Additional information

**How to cite this article:** Shlomai, H. & Fineberg, J. The structure of slip-pulses and supershear ruptures driving slip in bimaterial friction. *Nat. Commun.* 7:11787 doi: 10.1038/ncomms11787 (2016).
